# A novel partial volume correction method for accurate quantification of [^18^F] flortaucipir in the hippocampus

**DOI:** 10.1186/s13550-018-0432-2

**Published:** 2018-08-15

**Authors:** Emma E. Wolters, Sandeep S. V. Golla, Tessa Timmers, Rik Ossenkoppele, Chris W. J. van der Weijden, Philip Scheltens, Lothar Schwarte, Robert C. Schuit, Albert D. Windhorst, Frederik Barkhof, Maqsood Yaqub, Adriaan A. Lammertsma, Ronald Boellaard, Bart N. M. van Berckel

**Affiliations:** 1 Departments of Radiology & Nuclear Medicine, VU University Medical Center, PO Box 7057, 1007MB Amsterdam, The Netherlands; 2 Neurology & Alzheimer Center, VU University Medical Center, Amsterdam, The Netherlands; 3 Anaesthesiology, Amsterdam Neuroscience, VU University Medical Center, Amsterdam, The Netherlands; 4 Lund University, Clinical Memory Research Unit, Lund, Sweden; 5 Institutes of Neurology & Healthcare Engineering, UCL, London, UK

## Abstract

**Background:**

Off-target binding in the choroid plexus (CP) may cause spill-in of the tau PET tracer [^18^F] flortaucipir into the adjacent hippocampus region. The impact of this spill-in on hippocampal uptake was assessed using a novel partial volume correction method (PVC).

**Methods:**

PVC was performed on 20 [^18^F] flortaucipir dynamic PET scans (10 probable AD and 10 controls). Volumes of interest (VOIs) were defined for both hippocampus and CP. The correlation between hippocampal and CP distribution volume (V_T_), with and without PVC, was determined. Both anatomically defined and eroded VOIs were used.

**Results:**

For controls, the correlation between hippocampal and CP V_T_ was significantly reduced after using PVC along with an eroded VOI (*r*^*2*^ = 0.59, slope = 0.80 versus *r*^*2*^ = 0.15, slope = 0.15; difference: *p* < 0.05). The same was true for AD patients (*p* < 0.05).

**Conclusion:**

PVC together with an optimized hippocampal VOI resulted in effective reduction of CP spill-in and improved accuracy of hippocampal V_T_.

**Electronic supplementary material:**

The online version of this article (10.1186/s13550-018-0432-2) contains supplementary material, which is available to authorized users.

## Introduction

The PET tracer [^18^F]flortaucipir([^18^F]AV-1451) is a promising biomarker for in vivo assessment of tau pathology in AD [1]. However, cautious interpretation of [^18^F] flortaucipir data is warranted, especially in the hippocampus. Indeed, although tau pathology affects the hippocampus relatively early in the course of the disease [2], [^18^F] flortaucipir uptake in the hippocampus does not distinguish AD patients from controls [3, 4]. A possible explanation may be that the relatively low spatial resolution of PET leads to an underestimation of the signal in a small volume of interest (VOI) such as that of the hippocampus [4], especially in case of atrophy [3]. Resulting partial volume effects (PVE) may cause spill-in or spill-out to adjacent regions with higher or lower activity, respectively [5]. In the hippocampal region, this could also be the case as high off-target binding in the adjacent choroid plexus (CP) [6] may cause spill-in of [^18^F] flortaucipir [3, 7].

Partial volume correction (PVC) methods are used to correct for PVE. Previous studies used several different MR-based PVC methods [8–11] to process [^18^F] flortaucipir scans [7, 11–17]. Although some studies reported no significant effects of PVC on the correlation between tau uptake and cerebrospinal fluid (CSF) measures, cognition, and diagnostic accuracy [13, 15, 17], the specific impact of the CP signal on hippocampal [^18^F] flortaucipir uptake has yet to be evaluated.

In this study, PVC was performed using a method that combines Van Cittert (VC) iterative deconvolution (IDM) with highly constrained back-projection (HYPR) denoising. The combination of HYPR and VC IDM (Hypr-IDM-Hypr, HDH) was recently developed and validated [18] and allows for more accurate quantification of PET images. The purpose of the present study was to assess the impact of CP activity on quantification of hippocampal [^18^F] flortaucipir binding and to correct for spill-in using HDH PVC.

## Methods

### Participants

Ten patients with probable AD [19] and 10 cognitively healthy controls from the Amsterdam Dementia Cohort of the VU University Medical Center were included. Probable AD patients were only included if they had a positive [^18^F] florbetaben (amyloid-β) PET scan (visually read) and/or an AD-like CSF profile [19–21]. All subjects underwent the same study protocol as described before [22]. The study protocol was approved by the Medical Ethics Review Committee of the VU University Medical Center. The data were acquired for a prospective study that was focused on model evaluation.

### Data acquisition

All subjects underwent 3D-T1 weighted and FLAIR scans on a 3.0 Tesla MR scanner (Ingenuity TF PET/MR, Philips Medical Systems, Best, The Netherlands).

Dynamic PET emission scans were acquired using a Philips Gemini TF-64 PET/CT scanner. The protocol consisted of a dynamic scan of 130 min, after injection of 224 ± 18 MBq [^18^F] flortaucipir, with a 20-min break after the first 60 min. Each part of the scanning period started with a low-dose CT scan for attenuation correction [22].

Both continuous and manual arterial blood sampling were performed [22], to obtain a metabolite corrected plasma input function.

### Data analysis

#### Partial volume correction

A recently described combination of HYPR denoising and VC IDM was used to generate HDH PVC PET images (*18*). The VC IDM was used for enhancing the spatial resolution of the PET images. As the signal-to-noise ratio (SNR) also reduces with each iteration, HYPR was used to limit the decrease in SNR, thereby preserving image quality.

#### Volumes-of-interest

The second session PET scan was co-registered to the first session PET scan using VINCI (developed by S Vollmar) [23] for motion correction between the two sessions. Both T1-weighted and FLAIR MR images were co-registered to the summed PET images (5 to 29 frames were used) using VINCI software. Hippocampal VOI was defined in two different ways. First, the complete hippocampal VOI was extracted from the Hammers template [24] using PVElab [25] and the T1-weighted MR images. The automatic delineation is not perfect, and so even after PVC, it is possible that a relationship between choroid plexus and hippocampus might still persist because of the presence of the choroid plexus region voxels in the hippocampal VOI definition. Henceforth, for the second method, CP was defined manually on HDH [18] PVC PET images and the MR FLAIR image was used to crosscheck the VOI definition. Next, the complete hippocampus VOI was superimposed on the FLAIR image, and voxels from this VOI that were in close vicinity of CP were removed from the VOI definition, resulting in an “eroded” hippocampus VOI (Additional file 1: Figure S1). Only voxels where CP overlapped with the hippocampus were removed (~ 40% ± 10% of the total hippocampal voxels were removed). For both methods, regional time activity curves (TACs) were extracted by superimposing the VOIs onto all frames of the PET scans.

#### Kinetic analysis

TACs were analyzed by non-linear regression (NLR) using the two tissue compartment model with reversible kinetics and blood volume fraction parameter (2T4k_V_B_). It has previously been shown that this is the preferred model to describe in vivo kinetics of [^18^F] flortaucipir [22, 26, 27]. Regional volume of distribution (V_T_) was used as outcome measure.

#### Statistical analysis

Coefficients of determination (*r*^2^) and the slope of both complete and eroded hippocampal V_T_ with CP V_T_ were calculated. This analysis was performed with and without PVC. In addition, interaction effects between hippocampal and CP V_T_ were evaluated. The non-parametric Wilcoxon signed rank test was used to calculate the differences between methods. *p* < 0.05 was considered statistically significant. Statistical analysis was performed in the GraphPad Prism 7.

## Results

Ten controls (average age 67.7 ± 6.8 years, MMSE score 29.2 ± 0.6) and 10 probable AD patients (average age 63.9 ± 7.8 years, MMSE score 23.9 ± 3.1) were included. There was no significant difference in age between AD patients and controls (*p* < 0.05). Additional file 2: Figure S2 illustrates the time activity curves for choroid plexus and hippocampus VOIs (with and without PVC).

**Fig. 1:**
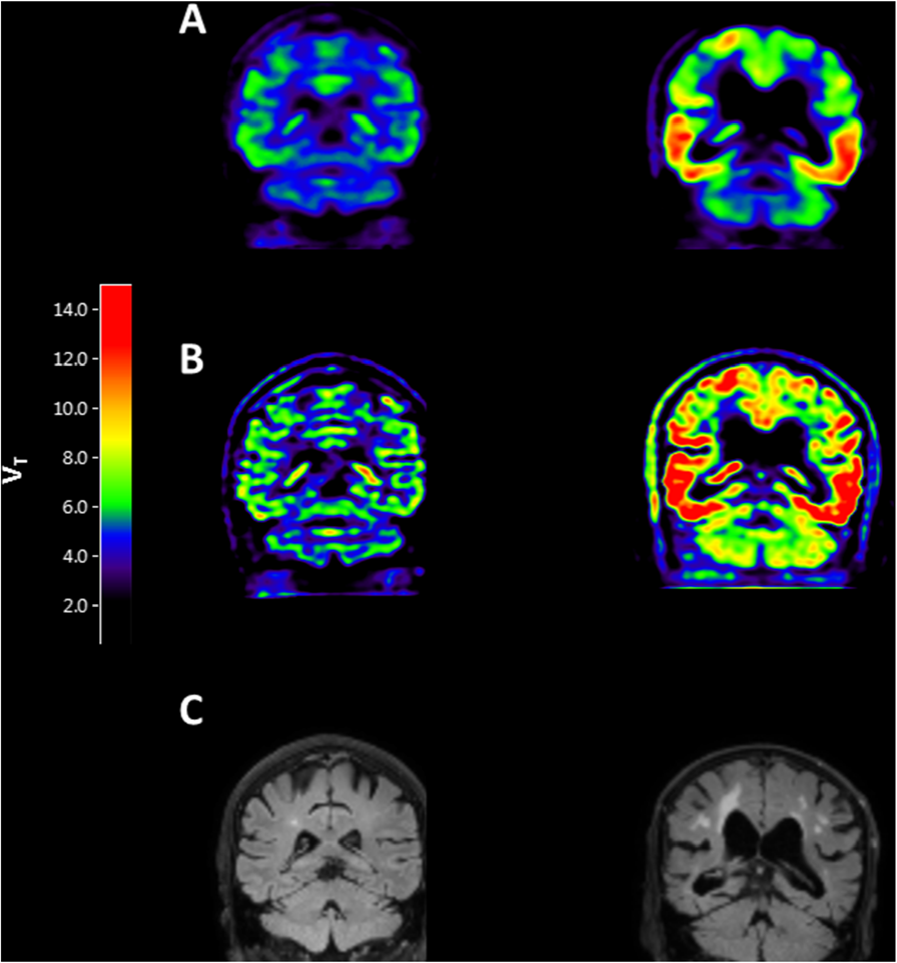
Coronal [^18^F] flortaucipir V_T_ images before (**a**) and after (**b**) HDH PVC and coronal MR flair (**c**) in a healthy control (left) and probable AD patient (right)

**Fig. 2:**
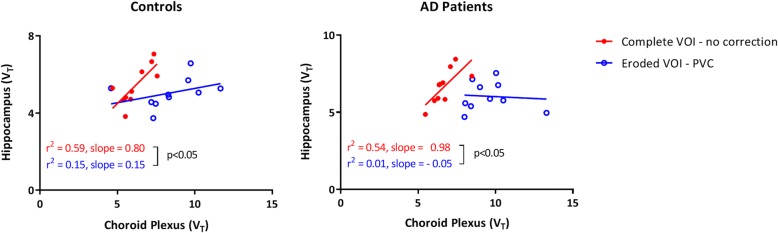
Scatter plots illustrating the relationship between hippocampus V_T_ and choroid plexus V_T_ before (red) and after (blue) using both PVC and an eroded hippocampal VOI for controls (left) and probable AD patients (right)

Before PVC, the relationship between the hippocampus and CP in controls decreased for an eroded VOI (*r*^*2*^ = 0.45, slope = 0.53) compared with the complete VOI (*r*^*2*^ = 0.59, slope = 0.80). After PVC, a further decrease was observed for both complete VOI (*r*^*2*^ = 0.29, slope = 0.27) and eroded VOI (*r*^*2*^ = 0.15, slope = 0.15). The relationship between hippocampus and CP uptake was significantly reduced when using an optimized VOI in combination with PVC (*p* for interaction < 0.05) (Fig. 2 and Additional file 3: Figure S3). An even stronger effect was observed in case of AD patients when using PVC in combination with the optimized hippocampal VOI (*r*^*2*^ = 0.54, slope = 0.98 without PVC versus *r*^*2*^ = 0.01, slope = − 0.05 with PVC; p for interaction < 0.05) (Figs. 1 and 2).

**Fig. 3:**
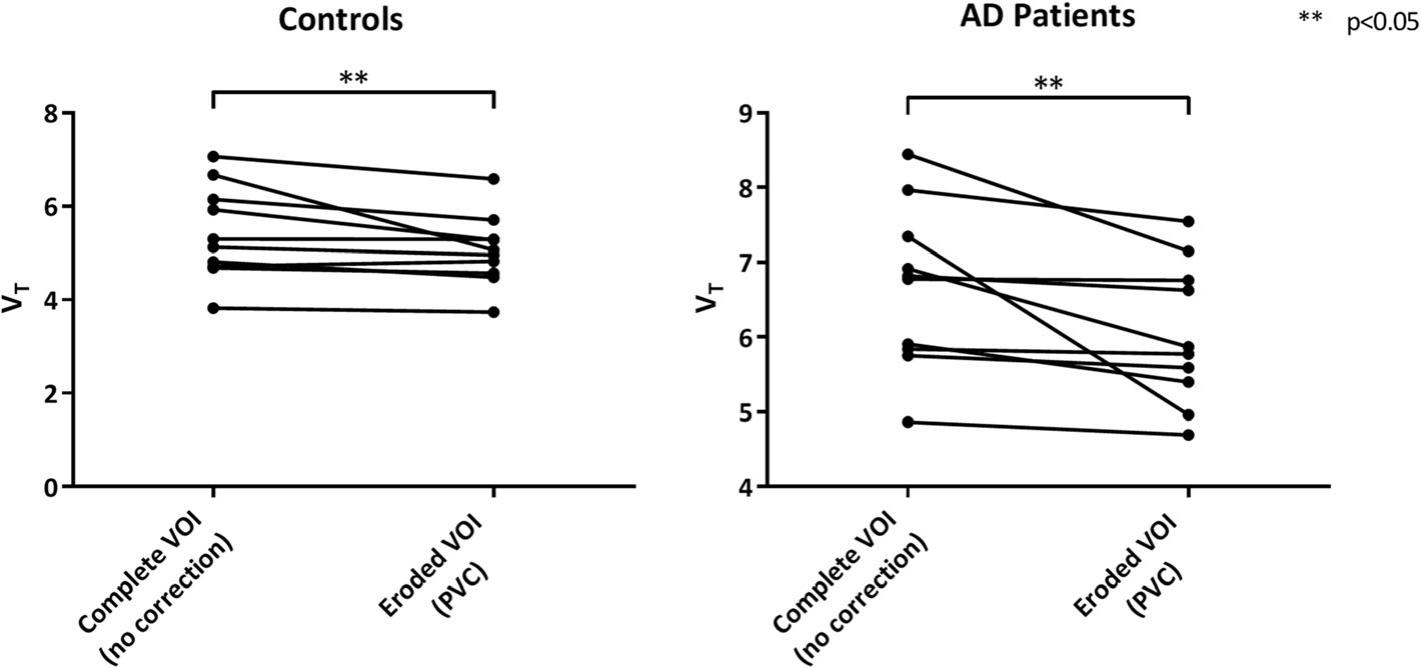
Spaghetti plots of hippocampus V_T_ before and after using both PVC and an eroded hippocampal VOI within controls (left) and probable AD patients (right)

In addition, there was a significant decrease in hippocampal V_T_ after using both PVC and eroded VOI compared with the uncorrected data (Fig. 3 and Additional file 4: Figure S4, *p* < 0.05). V_T_s obtained for the choroid plexus and hippocampus (complete and eroded) both before and after PVC for all the subjects included in the study (mean ± SD) are presented in the Additional file 5: Figure S5.

## Discussion

In order to improve the quantification of [^18^F] flortaucipir uptake in the hippocampus, a novel HDH PVC method was used together with an eroded hippocampal VOI to correct for possible spill-in effects from the CP. After PVC and optimizing hippocampal VOI, only a weak correlation remained between hippocampal V_T_ and CP V_T_, suggesting that spill-in from the CP was successfully reduced thus allowing for more accurate quantification of hippocampal [^18^F] flortaucipir uptake.

Wang et al. [16] showed that PVC [10] reduced the correlation between [^18^F] flortaucipir hippocampal and CP uptake (*r* = 0.39, *p* = 0.003 before PVC vs *r* = 0.14, *p* = 0.31 after PVC). Wang et al. seem to have mitigated the CP’s spill over without the use of hippocampal VOI erosion. Although the PVC implementation used by Wang et al. is not similar to the method used in this study, it can be argued that the relationship between choroid plexus and hippocampus is mitigated when applying PVC. With regard to the use of eroded hippocampal VOI, the possible reason for Wang et al. to not require erosion of hippocampal VOI could be that relationship present in their data between choroid plexus and hippocampus was not as strong (*r* = 0.39) as in our data (*r* = 0.76, combining both AD and controls). In case of a weak relation, possibly just PVC is sufficient; however, in case of a stronger relation, erosion might be necessary. Another point to note is that before PVC, no significant difference in the V_T_s was observed between the complete hippocampal VOI and eroded hippocampal VOI (Additional file 5: Figure S5). This suggests that the benefit of erosion was only observed along with the PVC. In addition, Schöll et al. [7] showed that PVC [8] caused a large increase in CP signal, indicating that spill-out from the CP could be reduced, potentially leading to a more accurate estimation of tau uptake in the adjacent hippocampus. Both studies suggest that PVC can be used to reduce spill-over effects from the CP. Nevertheless, both studies used MR-based PVC methods, and segmentation problems could result in a potential bias [10].

One of the strengths of the present study is that the VC IDM method leads to more accurate quantification of PET images and does not use MR scans for PVC, thereby avoiding potential segmentation and co-registration error. The addition of HYPR reduces the poor SNR of VC IDM, thus preserving image quality [18].

A potential weakness of this study is the fact that CP VOI was defined on PVC PET images rather than on MR images. However, CP activity could clearly be distinguished from that of surrounding tissue, as it was both visually and quantitatively much higher. Even then, a manual VOI definition is prone to error and inter-subject variability. In this study, the same researcher worked on the VOI definition to avoid inter-subject variability and to mitigate the error in VOI definition, and the manual VOIs of CP on PET PVC images were validated using MR FLAIR images. PET PVC-based VOI definitions showed good correspondence to the MR FLAIR images, suggesting that PVC PET images can be used for reliable definition of the CP VOI; however, an automatic or a semiautomatic VOI erosion method is warranted. Another limitation would be that there is no ground truth (no autopsy data available) to check the accuracy of the implementation. However, based on the presented analysis, it can be stated that the proposed methodology mitigates the spill-over effects of choroid plexus on hippocampus uptake. 

## Conclusion

The use of our new PVC method in combination with an optimized hippocampus VOI significantly reduces spill-in of CP activity into the hippocampus and improves accuracy of hippocampal V_T_.

## Additional files

Additional file 1: **Figure S1.** Illustrates the eroded hippocampal VOI definition on a T1 weighted MR scan of a subject (Sagittal slice). (TIF 680 kb)

Additional file 2: **Figure S2.** Time activity curves (TACs) of choroid plexus and hippocampus VOI (with and without PVC). In case of hippocampal VOI TACs for both complete and eroded VOI are presented. (TIF 475 kb)

Additional file 3: **Figure S3.** Relationship between the CP and hippocampus when using only erosion or PVC alone. (TIF 208 kb)

Additional file 4: **Figure S4.** Spaghetti plots of hippocampus complete VOI V_T_ (no corrections) and after using either erosion (no corrections) or PVC (complete VOI). (TIF 430 kb)

Additional file 5: **Figure S5.** Box plots (mean ± SD) for V_T_s obtained for the choroid plexus and hippocampus (using complete VOI or Eroded VOI) before and after PVC. (TIF 254 kb)
