# Supplementary figures and images for: A novel partial volume correction method for accurate quantification of [18F] flortaucipir in the hippocampus
Source: EJNMMI Res. 2018 Aug 15;8:79. doi: 10.1186/s13550-018-0432-2 (PMC6093830; doi:10.1186/s13550-018-0432-2)

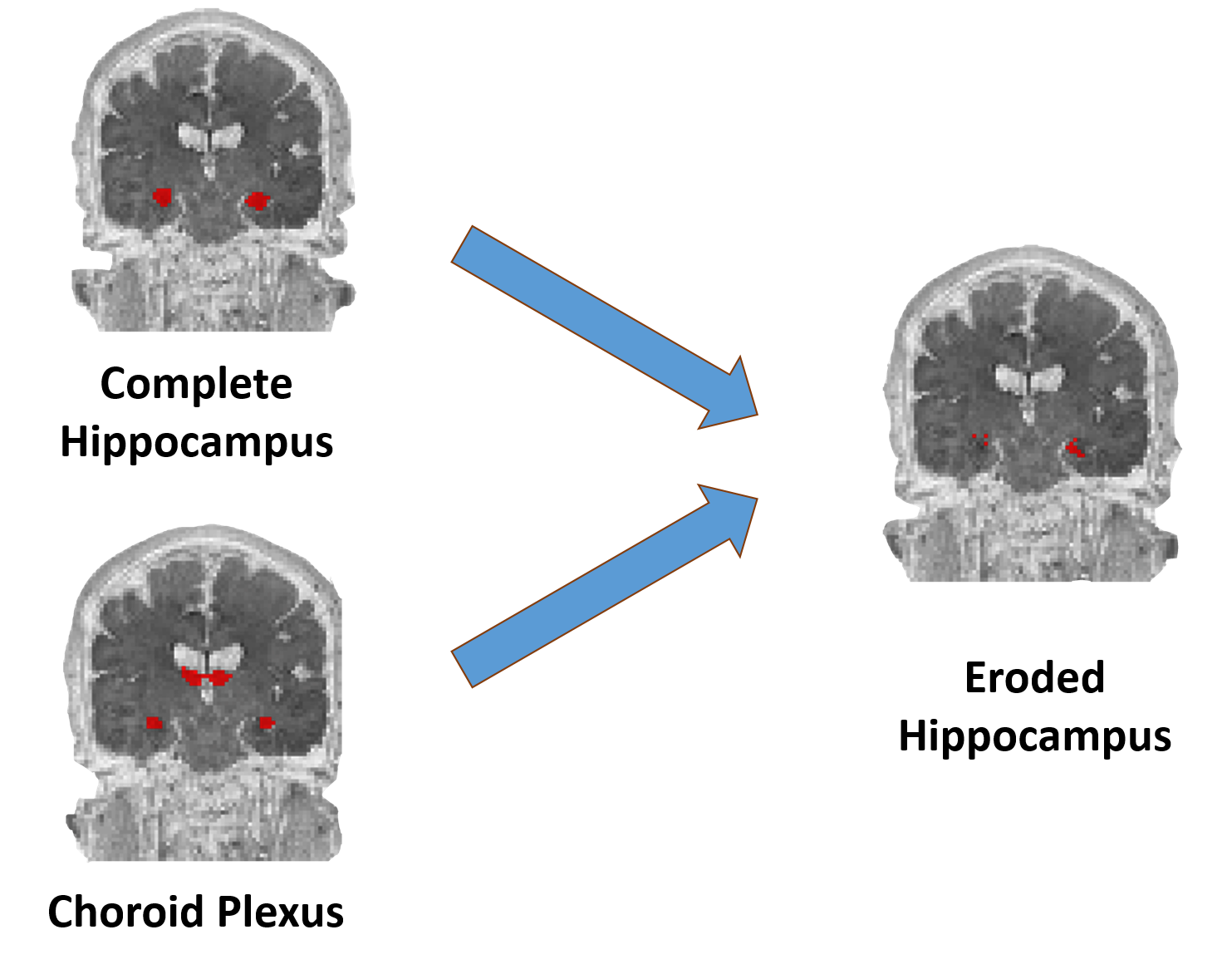

Supplement: Supplementary file 1 — Figure S1. Illustrates the eroded hippocampal VOI definition on a T1 weighted MR scan of a subject (Sagittal slice). (TIF 680 kb) [file 13550_2018_432_MOESM1_ESM.tif]

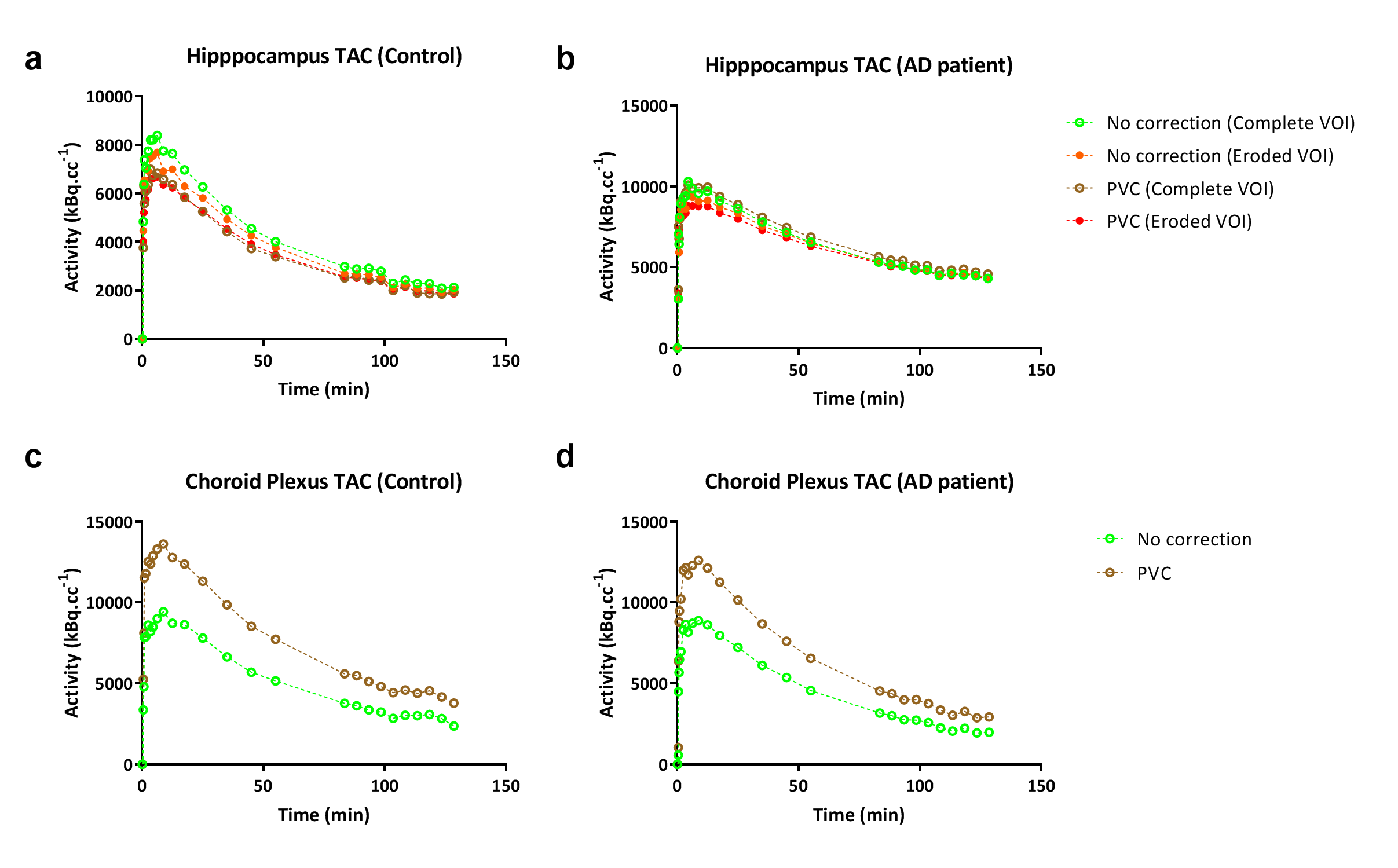

Supplement: Supplementary file 2 — Figure S2. Time activity curves (TACs) of choroid plexus and hippocampus VOI (with and without PVC). In case of hippocampal VOI TACs for both complete and eroded VOI are presented. (TIF 475 kb) [file 13550_2018_432_MOESM2_ESM.tif]

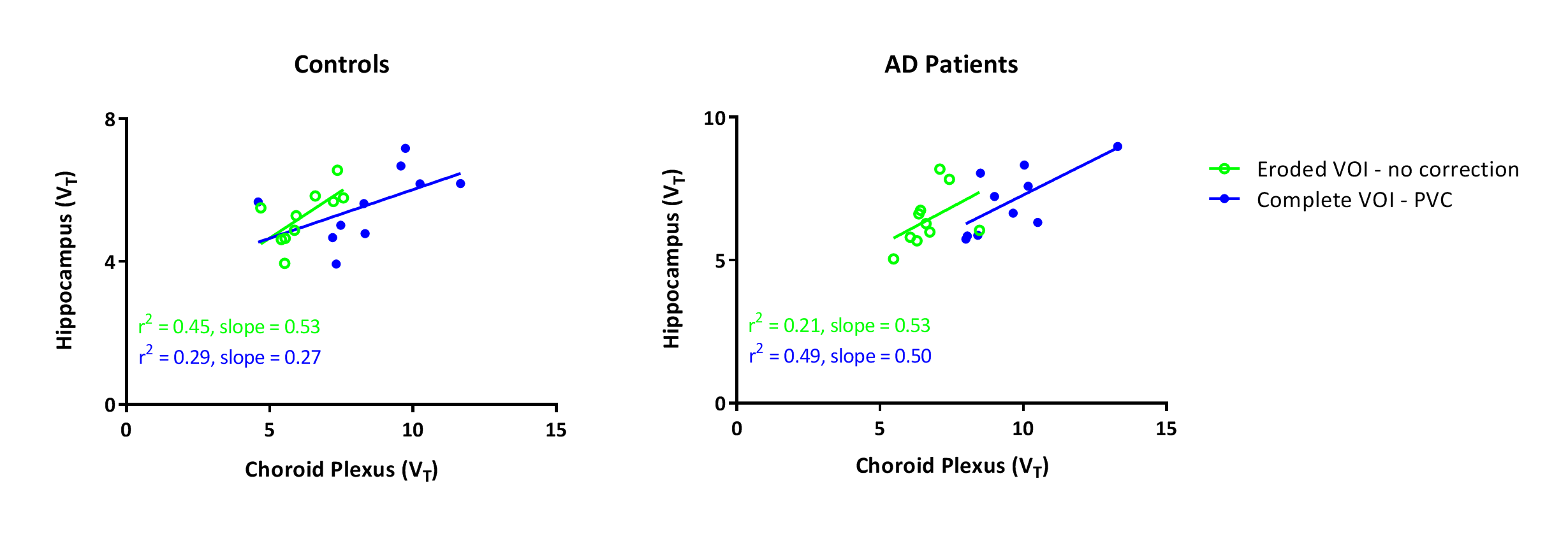

Supplement: Supplementary file 3 — Figure S3. Relationship between the CP and hippocampus when using only erosion or PVC alone. (TIF 208 kb) [file 13550_2018_432_MOESM3_ESM.tif]

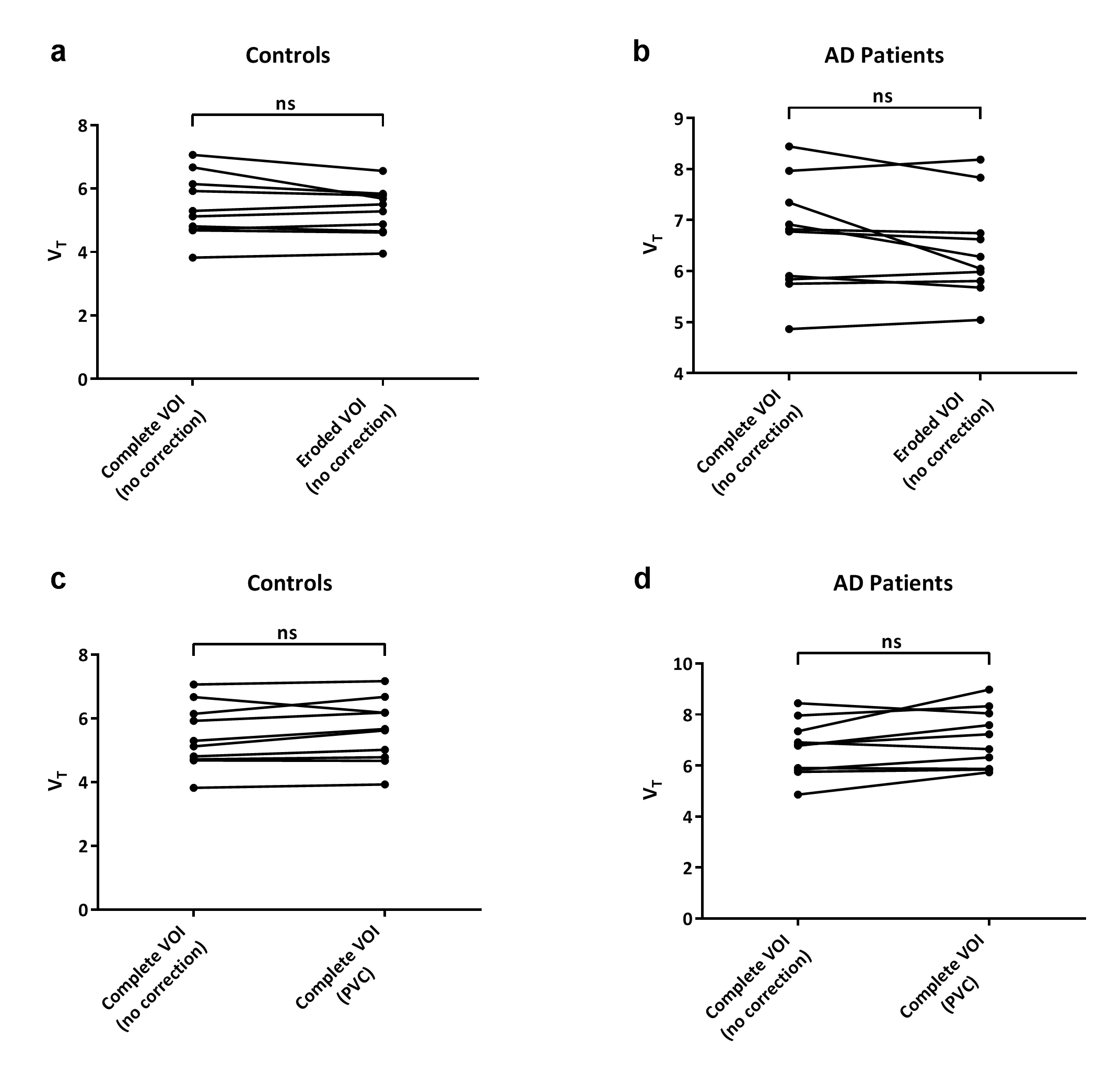

Supplement: Supplementary file 4 — Figure S4. Spaghetti plots of hippocampus complete VOI VT (no corrections) and after using either erosion (no corrections) or PVC (complete VOI). (TIF 430 kb) [file 13550_2018_432_MOESM4_ESM.tif]

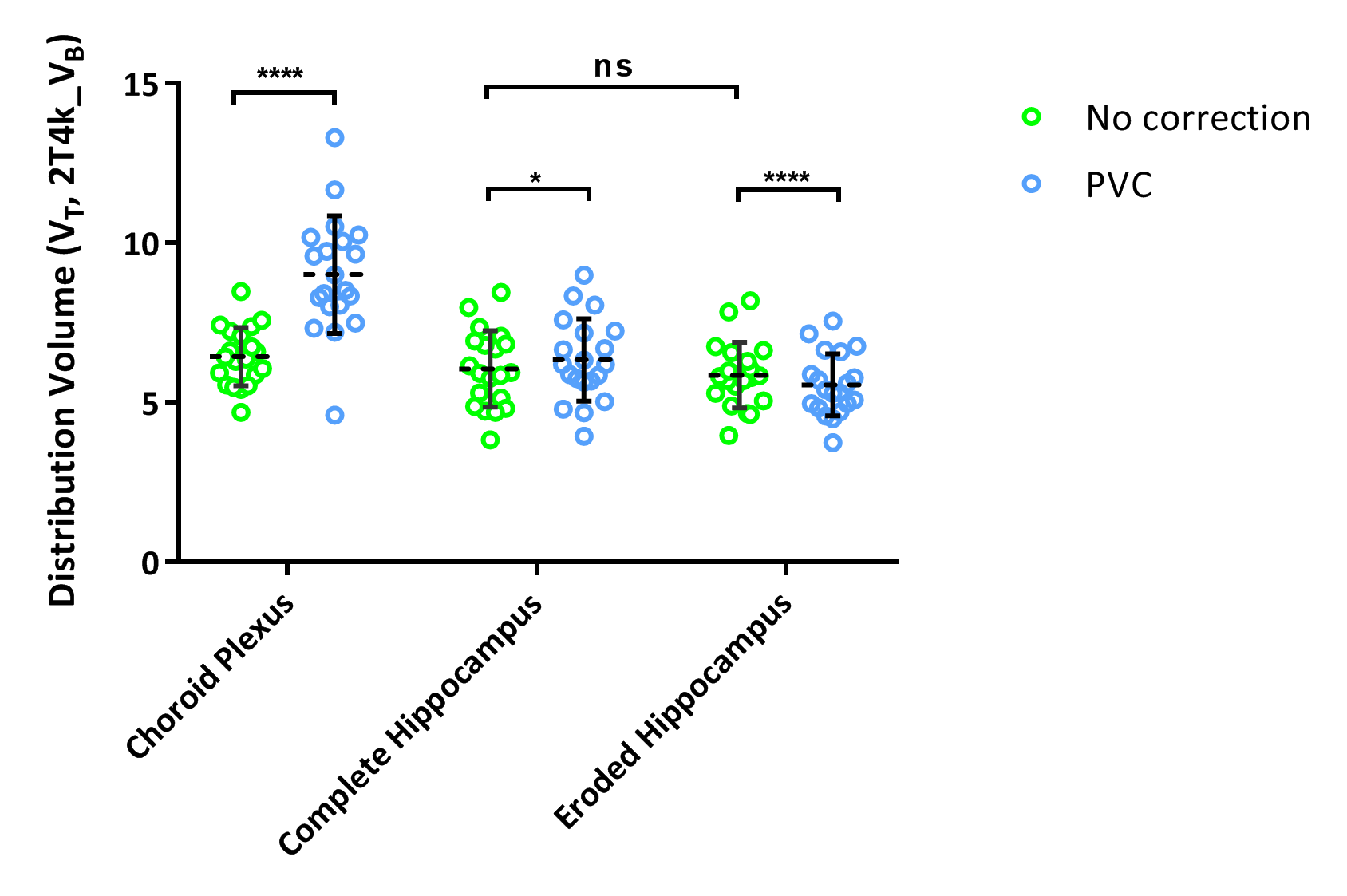

Supplement: Supplementary file 5 — Figure S5. Box plots (mean ± SD) for VTs obtained for the choroid plexus and hippocampus (using complete VOI or Eroded VOI) before and after PVC. (TIF 254 kb) [file 13550_2018_432_MOESM5_ESM.tif]
